# A Pilot Study of Twice-Weekly Group-Based Written Exposure Therapy for Veterans in Residential Substance Use Treatment: Effects on PTSD and Depressive Symptoms

**DOI:** 10.21203/rs.3.rs-4511374/v1

**Published:** 2024-07-19

**Authors:** Natalia Doren, Fang-Hsi Chang, Amanda Nguyen, Kevin R. McKenna, Derek D. Satre, Shannon Wiltsey-Stirman

**Affiliations:** University of California, San Francisco; University of California, Berkeley; University of California, Berkeley; VA Palo Alto Healthcare System; University of California, San Francisco; Stanford University

## Abstract

**Background:**

Posttraumatic stress disorder (PTSD) is highly comorbid with substance use disorders (SUDs), resulting in high prevalence of PTSD among individuals in residential SUD care. However, there is limited research on integrating trauma treatment into residential SUD care settings. The aim of the present project was to conduct an initial evaluation of the effects of group-based Written Exposure Therapy (WET) on PTSD and depressive symptoms that was integrated into programming for individuals in residential SUD treatment.

**Methods:**

Participants were 48 Veterans with comorbid PTSD-SUD from a 28-day residential SUD program at a Veterans Affairs Medical Center. Eligible participants were enrolled in 5 sessions of WET, delivered in twice-weekly in a group format. PTSD symptoms and depressive symptoms were assessed at each session with the Posttraumatic Stress Disorder Checklist, DSM-5 version (PCL-5) and the Patient Health Questionnaire (PHQ-9).

**Results:**

Of the enrolled sample of 48 participants, 92% (*n* = 44) completed 3 sessions, while 56% (*n* = 28) completed 5 sessions. Dependent samples t-tests showed significant reductions from baseline in PTSD symptoms within-persons at both the 3-session (*t*(43) = 4.77, *p* < .001, *d* = .72) and 5-session mark (*t*(27) = 4.36, *p* < .001, *d* = .82). In addition, there were significant reductions in depressive symptoms after 3 sessions (*t*(38) = 3.01, *p* < .01, *d* = .48) and after 5 sessions (*t*(23) = 2.97, *p* < .01, *d* = .61).

**Conclusion:**

Findings demonstrate that brief, group-delivered WET shows promise for addressing PTSD and depressive symptoms in residential SUD treatment. Results of the present evaluation could inform further efficacy testing and implementation of PTSD treatment into residential SUD settings.

## Introduction

A Pilot Study of Twice-Weekly Group-Based Written Exposure Therapy for Veterans in Residential Substance Use Treatment: Effects on PTSD and Depressive Symptoms

Posttraumatic stress disorder (PTSD) is highly comorbid with substance use disorders (SUDs). PTSD prevalence in individuals with SUD is about 30–40% in civilian samples (Back et al., 2000; Gielen et al., 2012; Kessler et al., 2005) and between 50–63% in Veteran samples (Roberts et al., 2015; Seal et al., 2011). Moreover, estimates suggest that up to 50% of individuals in residential care for SUDs have PTSD (Reynolds et al., 2005). Comorbid PTSD and SUD (PTSD-SUD) is associated with greater impairment, homelessness, and worse psychosocial functioning as well as lower income and lower likelihood of being married or partnered (Norman et al., 2018; Riggs et al., 2008; Simpson et al., 2019). Additionally, individuals with PTSD-SUD have higher rates of SUD relapse compared to those with either disorder alone (Bradizza et al., 2006; Kileen et al., 2015). Moreover, likelihood of SUD relapse in individuals with PTSD-SUD is linked to the severity of PTSD symptoms (Syan et al., 2020), underscoring the significance of addressing PTSD to facilitate recovery from SUD.

Despite the high prevalence of PTSD-SUD, the availability of evidence-based trauma treatment within residential SUD programs remains limited. A recent study found that only 16.6% of residential SUD treatment facilities in the US offer trauma treatment services (Spivak et al., 2022). Even within Veterans Affairs (VA) healthcare settings, where the majority of SUD treatment-seeking Veterans have comorbid PTSD (e.g., 63%; Seal et al., 2011), only 25% of residential VA SUD facilities offer PTSD treatment (Haller et al., 2019). Key barriers to implementation include limited time within a residential setting to deliver full-length evidence-based trauma protocols (e.g., 12-session prolonged exposure or cognitive processing therapy; Henslee et al., 2011); lack of clinical knowledge on how to integrate PTSD treatment into SUD care (Back et al., 2009); and lack of training in PTSD treatments (Kileen et al., 2015). Moreover, residential SUD programs are often understaffed (Im et al., 2015), further hindering the feasibility of offering a trauma protocol within the traditional individual (one-on-one) PTSD intervention framework. Thus, there is a need for identifying ways to integrate trauma treatment in residential SUD settings in a manner that is both effective and realistic.

Importantly, simultaneous PTSD and SUD treatment has been shown to be effective in addressing both PTSD symptoms and improving SUD outcomes. Meta-analytic evidence suggests that including trauma-focused cognitive behavioral treatment for PTSD concurrently with SUD treatment is linked to better PTSD and SUD outcomes 5–7 months later compared to SUD-only interventions (Roberts et al., 2015). In particular, exposure therapy is effective for addressing PTSD-SUD (Berenz et al., 2012; Simpson et al., 2021; Tripp et al., 2019). Notably, prior concerns in the field that exposure-based PTSD treatments would not be well-tolerated by those with SUDs have not been substantiated (Back et al., 2019; Schact et al., 2017; Simpson et al., 2017).

Unfortunately, there has been very little research on integrating PTSD treatment into SUD residential settings specifically—the majority of previous work on combined PTSD-SUD intervention has been conducted in outpatient settings. To date, only two randomized controlled trials s have been conducted testing the efficacy of trauma treatment in residential SUD care. Coffey et al. (2016) found that randomization to prolonged exposure therapy (PE) resulted in greater reduction in PTSD symptoms compared to controls in a community sample of residential SUD patients with PTSD. However, the study excluded individuals receiving medication assisted treatment, limiting generalizability. In addition, Beck et al. (2019) found that randomization to an intervention consisting of twelve 90-minute individual PE sessions was effective at reducing both PTSD and SUD symptoms in Veterans. While effective, delivering individual 90-minute PE sessions for all patients with PTSD in a residential SUD program may not be feasible in most residential settings, due to the high prevalence of PTSD-SUD and low staffing ratios (Im et al., 2015). Thus, there is a need for further research examining brief treatments that are effective and scalable in residential SUD care.

Written Exposure therapy (WET) is a brief trauma intervention that leverages traditional exposure techniques in a written format (Sloan & Marx, 2019). WET follows a 5-session sequence and primarily focuses on engaging in exposure by writing repeatedly about the index trauma event. Unlike other exposure-based treatments (e.g., prolonged exposure; cognitive processing therapy), WET does not include homework assignments and entails minimal discussion of the index trauma. Accordingly, WET may have several implementation advantages compared to other interventions, such as its brief format, the ability to conduct the therapy in a group setting while retaining confidentiality, and relatively low patient and provider burden, making it a promising approach for residential SUD settings.

A growing number of studies have been published on WET, primarily on individually delivered formats (Sloan & Marx, 2024). Evidence thus far suggests that WET is effective in treating PTSD (Sloan et al,. 2012). Moreover, WET has demonstrated non-inferiority to two well-established PTSD treatment models, cognitive processing therapy (Sloan et al., 2018; 2022) and prolonged exposure therapy (Sloan et al., 2023). In addition, WET has shown promise for addressing PTSD in patients and settings that face challenges in terms of time limitations. For example, WET evinced significant reductions in both PTSD and depressive symptoms in college students and fit well within their busy schedules (Morissette et al., 2017). One prior study evaluated the acceptability, feasibility, and initial effects of WET for residential SUD treatment (Schacht et al., 2023) and found that WET was feasible and acceptable, and resulted in significant reductions in PTSD symptoms. However, the study examined individually-delivered WET, and it is not known whether group-delivered WET would have similar effects.

Determining effects of group-based WET is important given the majority of psychotherapy treatment in residential SUD takes place in a group format (Wendt & Gone, 2018). One case study examined bi-weekly group WET for individuals in residential SUD treatment (Schumacher et al., 2023) and found that the intervention was associated with PTSD symptom reduction in all three cases. However, no studies to date have examined the impact of group-based WET on PTSD symptom reduction in residential SUD programs for Veterans with PTSD. Veterans have higher PTSD symptom severity, higher likelihood of having experienced multiple traumas, and greater rates of other mental health comorbidities compared to civilians (Wisco et al., 2014). Thus, examining whether WET results in PTSD symptom reduction in this complex patient population is needed to establish initial effects before commencing an efficacy study.

We conducted a pilot evaluation of group WET in a residential SUD program to examine feasibility, tolerability, and initial outcomes. The aim of the present project is to report on our evaluation of the effects of twice-weekly group-delivered WET on PTSD and depressive symptoms in a sample of Veterans with PTSD-SUD enrolled in a 28-day residential SUD treatment program. Groups were provided twice-weekly (as opposed to once-weekly, a more typical group therapy schedule) because residential SUD program stays are often of short duration, e.g., due to stepping up or down a level of care, leaving against medical advice (AMA) or other reasons. We chose to examine depressive symptoms in addition to PTSD symptoms because evidence suggests that exposure therapy for PTSD can also improve depressive symptoms (Brown et al., 2018), and that increases in negative affect and depressive symptoms predict relapse to SUDs (Witkiewitz & Boewn, 2010). Thus, it was anticipated that this pilot evaluation would provide valuable insight into the potential impact of the WET intervention on both PTSD and depressive symptoms.

## Method

### Treatment Setting

The present evaluation took place in a four-week (28-day) VA residential SUD program in Northern California. The standard treatment regimen on the unit includes individual case management, group psychotherapy, individual psychotherapy, medication assisted treatment, and psychiatric medication for co-occurring disorders. Specifically, group psychotherapy typically focuses on motivational interviewing, relapse prevention, cognitive behavioral therapy, and building skills to support recovery. Skills groups draw from a dialectical behavior therapy (DBT; Linehan, 1993) framework, including four specific modules addressing each DBT theme—mindfulness, distress tolerance, emotion regulation, and interpersonal effectiveness. In addition, individual psychotherapy is typically tailored to meet unique needs of each patient, primarily focused on motivational interviewing and cognitive behavioral therapy to address SUD. No evidenced-based PTSD-specific programming outside of the WET group was offered.

### Participants

WET group participants were Veterans who were enrolled in a 28-day residential SUD treatment program at a VA medical center between April 2023-Sept 2023. Patients in the program who had a diagnosis of PTSD at intake (assessed through clinician interviews) were invited to join the WET group by staff members. Staff confirmed interest in participation and scheduled an orientation session. Eligibility criteria for the group were (1) sufficient memory of the index trauma to write narratives about it; (2) had at least 2 weeks remaining at the facility (to ensure sufficient time to complete the group); (3) did not have severe suicidality and/or severe psychotic symptoms.

### Procedures

The project was submitted to the local Institutional Review Board IRB and was determined to be a quality improvement project that is exempt from further IRB oversight, given the nature of the treatment offered to patients as part of routine care. Eligible group participants completed a 1-hour individual orientation session to discuss participation in the group, confirm eligibility and interest, and determine the index event. Patients then engaged in WET group therapy for 5 sessions. Sessions were administered twice weekly for 1-hour each. All sessions were provided by a staff member. After each session, participants completed PTSD and depressive symptom measures, as detailed below, and aligned with routine quality of care measures used in the treatment setting.

#### Orientation Session.

Participants were enrolled in the residential SUD program for at least 3 days before starting the WET group to allow time to acclimate and avoid acute intoxication or withdrawal effects. Eligible patients met with a staff member to orient the participant to the group procedures, provide psychoeducation about trauma treatment, and conduct a detailed assessment of trauma history, following procedures outlined in the WET protocol (Sloan & Marx, 2019). If patients reported at least one DSM-5 criterion A event, the clinician worked with the patient to identify an appropriate index trauma to be the focus of the WET group sessions. Appropriateness was determined by (1) having a clear memory of the event, (2) the event was causing significant PSTD symptoms (e.g., was the subject of PTSD flashbacks and related to significant avoidance of trauma reminders), and (3) the participants’ stated willingness to work on the event. Following the trauma assessment, the staff member assessed the potential for comorbidities to interfere with treatment. Finally, the staff member then provided an opportunity for the patient to ask any questions they may have about participating in the group along with identifying and problem-solving any roadblocks to participation (e.g., coordinating medical appointments).

#### Written Exposure Therapy (WET).

The WET intervention was administered as per procedures outlined in Sloan and Marx (2019). The intervention was delivered by a staff member with a PhD in psychology who had prior training in the WET protocol through a combination of didactics, supervision, and consultation. Session 1 of WET involved psychoeducation on trauma and exposure therapy, reviewing what to expect out of treatment, and laying out the rationale for WET. Participants also learned about how to monitor distress during exposure using the verbally administered subjective units of distress (SUDS) rating scale so that they could provide distress ratings pre- and post-exposures. Following this, individuals were instructed to write for 30 minutes about the index event, focusing on specific sensory details (e.g., what they saw, heard, smelled), as well as their thoughts and feelings about the trauma (e.g., “I was frozen with fear”) as they remember it in the present moment. After their first exposure, they received psychoeducation on avoidance and the possibility of symptom exacerbation during treatment. After Session 1 and before Session 2, the staff clinician reviewed their written exposures and provided individualized feedback to each patient privately, such as whether they omitted emotions, thoughts, and sensory details, the length of the account, and whether the patient wrote about the agreed upon index trauma. In addition, safety monitoring was performed throughout by staff during and after each session by reviewing the content of written exposures to check for indications of risk (e.g., suicidal ideation) and during individual check-ins as part of routine care on the unit. In Session 2, participants repeated the written exposure process from Session 1, while incorporating any feedback on the exposure process from the staff study member. In Sessions 3–5, participants receive additional instructions to write about how the trauma experience has impacted various aspects of their lives, including changes in their lifestyles, perspectives, the meaning of life, and their relationship with others, in addition to narrating the index event as they did previously. Following 30 minutes of written exposure, the remainder of the sessions were used to allow participants to verbally reflect on the process (but not content) of the exposure experience with the group if they choose to do so. At each session, participants provided verbal SUDS ratings before and after exposure.

### Measures

#### PTSD symptoms.

As is standard in WET, PTSD symptoms were assessed by clinical staff before each session with the validated PTSD Checklist for DSM-5 (PCL-5 weekly version; Blevins et al., 2015) via paper-and-pen measures that were entered into the healthcare record by clinicians. The PCL-5 is a 20-item measure, with possible range 0–80, and assesses PTSD symptoms on a scale of 0 (not at all bothered) to 4 (extremely bothered). A score of 31 or higher indicates probable PTSD. Total scores were obtained for each patient at each session from the electronic health record. Reliability for the current sample is not available due to a lack of item-level data from patient health records.

#### Depressive symptoms.

Depressive symptoms were also assessed by clinical staff before each session with the validated Patient Health Questionnaire (PHQ-9; Kroenke et al., 2001) via paper-and-pen. The PHQ-9 is a 9-item measure with possible range from 0–27. Scores of 0–9 indicate mild-moderate symptoms, 9–19 indicate moderate-severe symptoms, and 20–27 indicating high severity depression. Total scores were obtained for each patient at each session and entered into the healthcare record by clinicians. Reliability for the current sample is not available due to a lack of item-level data from patient health records.

### Data Analysis

All analyses were conducted in *R* Version 4.1.0 and *RStudio* Version 1.4.17 (R. Core Team, 2021). Descriptive statistics were computed using the *psych* package (Revelle et al., 2023) and *tidyverse* package (Wickham et al., 2019). Dependent samples t-tests were conducted to examine change in symptoms from Session 1–3 and Session 1–5 using base *R*. Effect sizes were calculated manually using the formula for dependent samples Cohen’s D = M_post_−M_pre_ / SD (M_difference_). All data and code are available on OSF: https://osf.io/4hkvz/?view_only=5e90af12780142ed907c1e5223a91a70.

## Results

### Sample Characteristics

Group participants were 48 veterans that ranged in age from 28 to 73 years (*M* = 46.29, *SD* = 13.19), and were predominantly men (87.5%). Race/ethnicity was obtained from the electronic health record as follows: White (58.3%, *n* = 28), Hispanic/Latine (22.92%, *n* = 11), Black (18.75%, *n* = 9), Asian (6.25%, n = 3) and other (6.25%, *n* = 3). Patients’ primary SUD diagnoses at intake (obtained from chart reviews) were as follows: Alcohol Use Disorder (79.17%; *n* = 79.17%), Stimulant Use Disorder (50%; *n* = 24), and Opioid Use Disorder (18.75%; *n* = 9). In addition, patients had a variety of comorbid mental health diagnoses other than PTSD, primarily Major Depressive Disorder (45.83%; *n* = 22), personality disorders (16.67%; *n* = 8), ADHD (14.58%; *n* = 7) and Substance-Induced Mood Disorder (10.42%; *n* = 5; (Table 1).

### Retention

**Figure 1:**
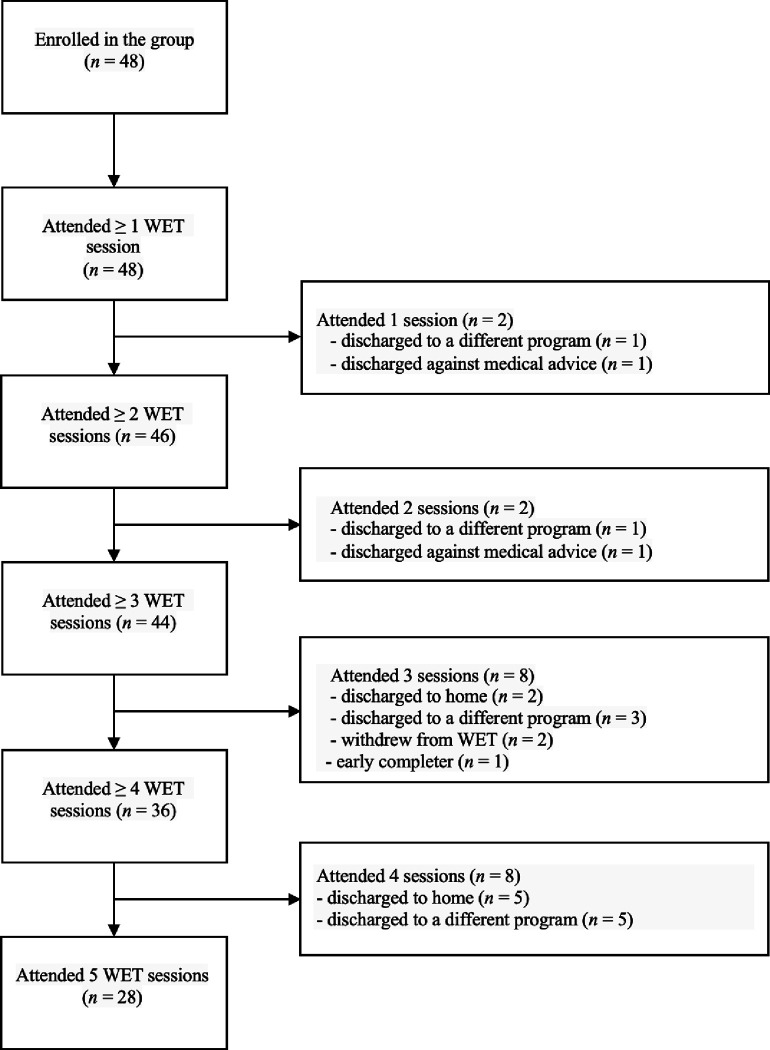
Participant flow diagram with reasons for dropout. *Note*. WET = written exposure therapy.

Figure 1 presents a patient flow diagram indicating *n* at each session and reasons for discharge. All group participants completed at least one WET session (100%; *n* = 48). Ninety-three percent (*n* = 45) completed three sessions, 75% (*n* = 36) completed four sessions, and 58% (*n* = 28) completed all five sessions. The primary reason for completing less than five sessions was discharge to home (14.5%; *n* = 7), discharge to another facility (12.5%; *n* = 6), and leaving the program against medical advice (0.06%; *n* = 3). In addition, two participants withdrew from the group due to avoidance or preference (0.04%; *n* = 2); one was an early completer (achieved symptom remission after 3 sessions and opted not to continue beyond that).

### PTSD Symptom Change

**Figure 2:**
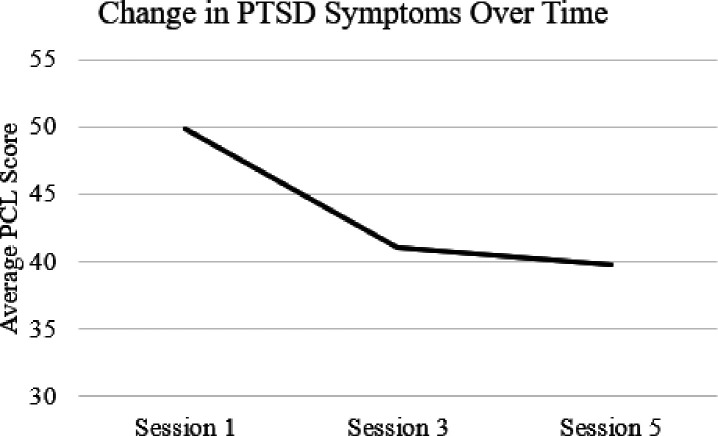
Average PTSD symptom scores across the entire sample from Session 3 through Session 5. PCL-5 = PTSD Checklist, DSM-5 version.

Figure 2 depicts the average PCL-5 scores at sessions one, three, and five across the entire sample. Results of dependent samples t-tests (Table 2) showed significant within-person reductions in PTSD symptoms from Session 1 to Session 3 (*t*(44) = 4.77, *p* < .001). The average reduction across the entire sample about 8.4 points. In addition, there were significant reductions in PTSD symptoms from Session 1 to Session 5 (Table 3; *t*(28) = 4.36, *p* < .001), with an average reduction of 13 scale points. Effect sizes were moderate for Session 1–3 change (*d* = 0.72) and large for Session 1–5 change (*d* = 0.82).

### Depression and PTSD Symptom Change

**Figure 3:**
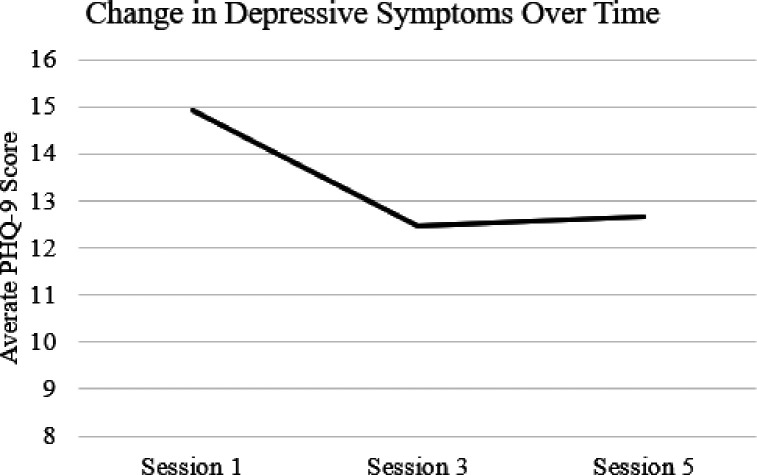
Average depressive symptoms across the entire sample from Session 3 through Session 5. PHQ-9 = patient health questionnaire, 9-item version.

Figure 3 depicts the average PHQ-9 scores at sessions one, three, and five across the entire sample. Results of dependent samples t-tests (Table 2) showed significant within-person reductions from Session 1 to Session 3 (*t*(39) = 3.01, *p* = .005). The average reduction across the entire sample of around three scale points. In addition, there were significant reductions in PTSD symptoms from Session 1 to Session 5 (*t*(24) = 3.01, *p* = .006), with an average reduction of 4 points. Effect sizes were small to moderate for both timepoints, with Session 1–5 change (*d* = 0.61) demonstrating a larger effect size compared to Session 1–3 change (*d* = 0.48).

### Remission from PTSD as per PCL-5 scores

Additional sub-analysis of change in PTSD symptoms amongst those who had PCL score ≥ 32 at the start of treatment to determine rates of remission after three sessions and five sessions, respectively. Among those that completed three sessions and had initial PCL scores of ≥ 32 (*n* = 41), 22% (*n* = 9) achieved remission from PTSD as assessed by PCL-5 scores ≤ 32. Amongst individuals that that completed five sessions and had initial PCL score ≥ 32 (*n* = 25), 32% (*n* = 8) achieved remission from PTSD as assessed by PCL-5 scores shifting from ≥ 32 to ≤ 32.

## Discussion

This report of a single-arm pilot evaluation represents the first investigation of the effects of WET on PTSD symptoms and depressive symptoms in Veterans undergoing residential substance use treatment. Findings suggest that twice-weekly group-delivered WET resulted in significant reductions in PTSD symptoms with moderate effect sizes in as little as three sessions, and a large effect size after five sessions. Moreover, WET had additional benefits in terms of reducing depressive symptoms after both three sessions and five sessions, with small to moderate effect sizes. While causality cannot be inferred from this nonrandomized pilot, findings contribute to the growing evidence base on WET and suggest that WET is a promising approach for addressing PTSD in a comorbid SUD population in a residential setting, where time and resources may be limited to conduct a full-length exposure-based therapy protocol.

Findings with respect to changes in PTSD symptoms showed that participants who completed three sessions experienced significant decreases in symptoms (8.4 scale points on average), while those who continued to complete the five sessions experienced even greater reduction (average of 13 points). Our findings add to a growing body of work on the effects of WET for those with PTSD (Sloan & Marx, 2024) and PTSD-SUD (Schact et al., 2023; Schumacher et al., 2023), demonstrating that effects may generalize beyond case studies and to Veterans in residential SUD treatment settings. At the same time, while results are encouraging with respect to symptom reduction, criteria for clinically significant change as established in prior work (e.g., Marx et al., 2022; 15 point reduction in PCL scores) were not met in this small pilot evaluation. Thus, further refinements to WET may be warranted to enhance patient outcomes amongst those with PTSD-SUD. For example, tailoring WET prompts to address specific needs of a PTSD-SUD population, such as by incorporating consideration of SUD-specific impacts of trauma into written exposure, would be a fruitful area of future investigation. In addition, level of symptom change was slightly lower than that found in a RCT of individual WET that examined change using CAPS scores (Sloan et al., 2023). Findings align with prior work on cognitive processing therapy that suggests group efficacy is generally lower than that of individual treatment (Resick et al., 2017). Nevertheless, our findings add to a growing body of work on the effects of WET for those with PTSD (Sloan & Marx, 2024) and PTSD-SUD (Schact et al., 2023; Schumacher et al., 2023), demonstrating that findings of symptom reduction may generalize beyond case studies and to Veterans in residential SUD treatment settings.

For depressive symptoms, changes were statistically significant with small to moderate effect sizes (*d* = .48 for three sessions; *d* = .61 after five sessions). Despite this, total average change in depressive symptom scores did not meet established thresholds for clinically significant change (5 scale points; Kroenke, 2012). Specifically, after three sessions, there was a 3 point change while 5 sessions resulted in an average reduction of 4 scale points. Thus, while group WET may have some impact on reducing depressive symptoms, larger effects were seen for PTSD symptoms overall. These findings are consistent with the primary focus of exposure therapy being to address PTSD symptomatology, with reductions in depressive symptoms often being an additional benefit, but not necessarily the target of treatment (Brown et al., 2015). In addition, it is possible that those with higher depressive symptoms at Session 1 saw greater reductions compared to the average; future work with larger samples could investigate this directly through moderation analyses. Nevertheless, demonstrated decreases in depressive symptoms is encouraging for future study.

Moreover, 22% of participants who completed three sessions and 32% of participants who completed five sessions achieved remission from PTSD as per the PCL-5 standard cutoffs. While findings should be interpreted with caution given the small sample size, examining individual effects underscores the promise of brief WET for addressing PTSD in comorbid populations in a very brief time frame with a potentially large payoff.

Overall, findings suggest that twice-weekly group-delivered WET is a promising approach for addressing PTSD treatment in PTSD-SUD populations in residential treatment settings. The fact that the intervention could be completed in as little as 2.5 weeks with biweekly sessions and in a group context makes it particularly appealing for implementation in residential SUD programs that are often understaffed, have high rates of attrition and turnover, and limited time to complete the intervention after assessments are performed (Im et al., 2015).

### Strengths, Limitations, and Future Directions

The present evaluation has several notable strengths, including examining the effects of the WET intervention in a real-world setting with the patient population for which the intervention is intended, conducting the intervention with a broad range of patients with complex comorbidities, and the use of psychometrically validated measures to assess PTSD and depressive symptoms. Moreover, findings add to the scant literature on the integration of PTSD treatment in residential SUD care. However, results should be interpreted with caution in light of several limitations. As a single-arm pilot, the lack of a control group precludes the ability to establish causal relationships between the intervention and symptom outcomes. Given that patients were undergoing treatment for SUD, it is possible that improvements occurred regardless of WET participation, such as due to other interventions they received while on the unit (e.g., substance use medications; case management). However, prior work suggests that SUD treatment alone does not by itself aid in reducing PTSD symptoms (Roberts et al., 2022; Simson et al., 2017). Future studies should employ random assignment to establish the efficacy of WET in comparison to usual care. Assessing outcomes over a longer follow-up period would also allow investigation of whether intervention can be sustained over time.

We did not assess the effects of the intervention on other mental health outcomes (e.g., anxious symptoms) nor in relation to substance use. We opted for this approach to limit participant and clinician burden and because the aims were to establish initial effects on PTSD primarily. Nevertheless, in future work, establishing additional benefits of the intervention on SUD-related outcomes would be valuable. Moreover, assessing putative mechanisms of the WET intervention is an exciting area of future investigation—e.g., whether WET could reduce avoidance of trauma triggers.

Limits on generalizability include the small sample size, specific population (Veterans, mostly male), and setting (the specific residential SUD treatment). Accordingly, testing the intervention not only in larger samples with random assignment, but also across other patient populations and treatment settings would be important to investigate in future work. At the same time, the target patient population and setting in this evaluation makes the work more applicable to future studies within these settings and populations.

Limitations notwithstanding, findings lay the groundwork for future work integrating PTSD treatment into residential SUD programs and suggests that WET may be a promising approach for addressing PTSD-SUD in a manner that is feasible within the inpatient context. Future work with larger samples should assess effect heterogeneity (i.e., treatment moderators) to examine “what works best for whom” with respect to PTSD-SUD treatment in residential SUD programs.

## Conclusion

The present study contributes valuable initial findings regarding the benefits of brief, group-based PTSD treatment for Veterans in residential substance use treatment. Results suggest that twice-weekly group-delivered WET in residential SUD settings may provide PTSD and depressive symptom relief in as little as three sessions. Offering WET to individuals with PTSD in residential SUD treatment programs may provide an important tool for addressing PTSD-SUD and could encourage uptake given its relatively lower provider burden compared to traditional, more extensive exposure therapy protocols. The present work sets the stage for further investigations and intervention developments to address the complex challenges of treating PTSD-SUD.

## Figures and Tables

**Table 1:** Demographic and Clinical Characteristics of WET Group Participants (N = 48).

	N or Mean	% or SD
Age	46.29	13.19
**Gender**		
Man	42	87.50%
Woman	4	8.33%
Other	2	4.17%
**Race & Ethnicity**		
White	28	58.33%
Latine	11	22.92%
Black	9	18.75%
Asian	3	6.25%
Other	3	6.25%
**Employment Status**		
No employment	43	89.58%
Active Employment	5	10.42%
**Mental Health Diagnoses**		
PTSD	48	100%
Major Depressive Disorder	22	45.83%
Personality Disorder	8	16.67%
ADHD	7	14.58%
Substance-induced Mood Disorder	5	10.42%
Schizoaffective Disorder	3	6.25%
Substance-induced Psychotic Disorder	3	6.25%
Generalized Anxiety Disorder	2	4.17%
Gender Dysphoria	1	2.08%
Bipolar II	1	2.08%
Eating Disorder	1	2.08%
**Primary SUD Diagnoses**		
Alcohol Use Disorder	38	79.17%
Stimulant Use Disorder	24	50%
Opioid Use Disorder	9	18.75%

*Note.* ADHD = attention deficit hyperactivity disorder. PTSD = post-traumatic stress disorder. Demographics and diagnoses were based on chart review obtained from electronic health records (EHR). PTSD diagnoses were confirmed via clinician interview, as indicated in the method.

**Table 2:** Dependent samples t-tests examining change in symptoms, Session 1 to 3 (n = 44).

	Session 1		Session 3		Session 1 vs. 3			
	*M*	*SD*	*M*	*SD*	*t*	*df*	*p*	Cohen’s *d*
PCL-5	49.8	15.3	41.1	18.5	4.77***	43	< .001	0.72
PHQ-9	14.9	6.15	12.5	6.14	3.01**	38	0.005	0.48

*Note.* PCL-5 = PTSD Checklist, DSM-5 version. PHQ-9 = patient health questionnaire, 9-item version.

*** *p* < .001

** *p* < .01.

**Table 3:** Dependent samples t-tests examining change in symptoms, Session 1 to 5 (n = 28).

	Session 1		Session 5		Session 1 vs. 5			
	*M*	*SD*	*M*	*SD*	*t*	*df*	*p*	Cohen’s *d*
PCL-5	52.18	15.21	39.79	19.64	4.36***	27	< .001	0.82
PHQ-9	16.07	6.57	12.67	5.90	3.01**	23	0.006	0.61

*Note.* PCL-5 = PTSD Checklist, DSM-5 version. PHQ-9 = patient health questionnaire, 9-item version.

*** *p* < .001

** *p* < .01.
